# Supplementary material for: Nasopharyngeal carriage of Streptococcus pneumoniae among children <5 years of age in Indonesia prior to pneumococcal conjugate vaccine introduction
Source: PLoS One. 2024 Jan 11;19(1):e0297041. doi: 10.1371/journal.pone.0297041 (PMC10783721; doi:10.1371/journal.pone.0297041)
Supplement: S1 Fig — (PPTX) [file pone.0297041.s001.pptx]

## Slide 1
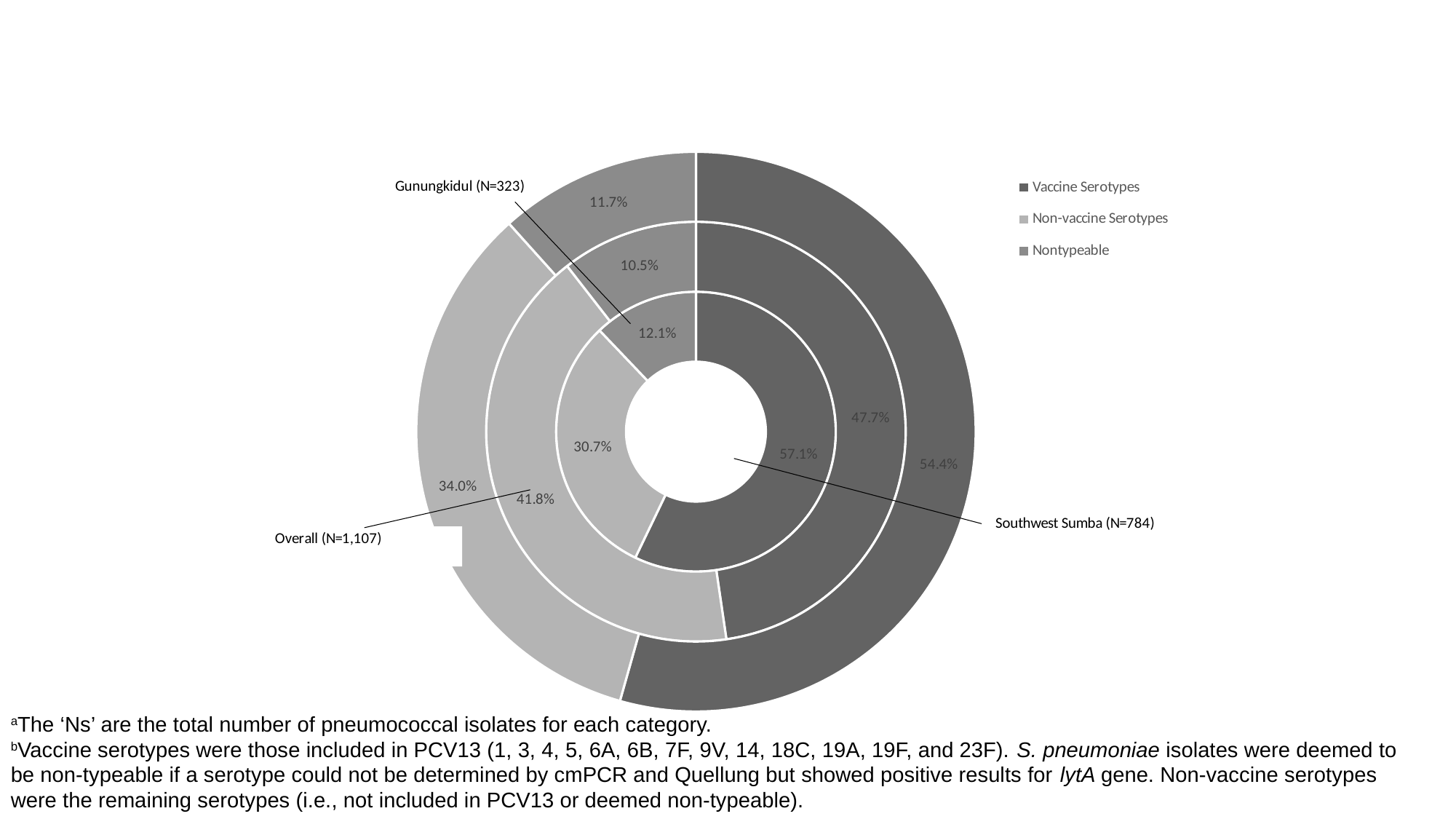

### Chart
| Category | Southwest Sumba | Gunungkidul | Overall |
|---|---|---|---|
| Vaccine Serotypes | 0.571428571428572 | 0.476780185758514 | 0.543812104787714 |
| Non-vaccine Serotypes | 0.307397959183673 | 0.417956656346749 | 0.339656729900632 |
| Nontypeable | 0.121173469387755 | 0.105263157894737 | 0.116531165311653 |aThe ‘Ns’ are the total number of pneumococcal isolates for each category.
bVaccine serotypes were those included in PCV13 (1, 3, 4, 5, 6A, 6B, 7F, 9V, 14, 18C, 19A, 19F, and 23F). S. pneumoniae isolates were deemed to be non-typeable if a serotype could not be determined by cmPCR and Quellung but showed positive results for lytA gene. Non-vaccine serotypes were the remaining serotypes (i.e., not included in PCV13 or deemed non-typeable).
