# Supplementary material for: Nasopharyngeal carriage of Streptococcus pneumoniae among children <5 years of age in Indonesia prior to pneumococcal conjugate vaccine introduction
Source: PLoS One. 2024 Jan 11;19(1):e0297041. doi: 10.1371/journal.pone.0297041 (PMC10783721; doi:10.1371/journal.pone.0297041)
Supplement: S3 Fig — (PPTX) [file pone.0297041.s003.pptx]

## Slide 1
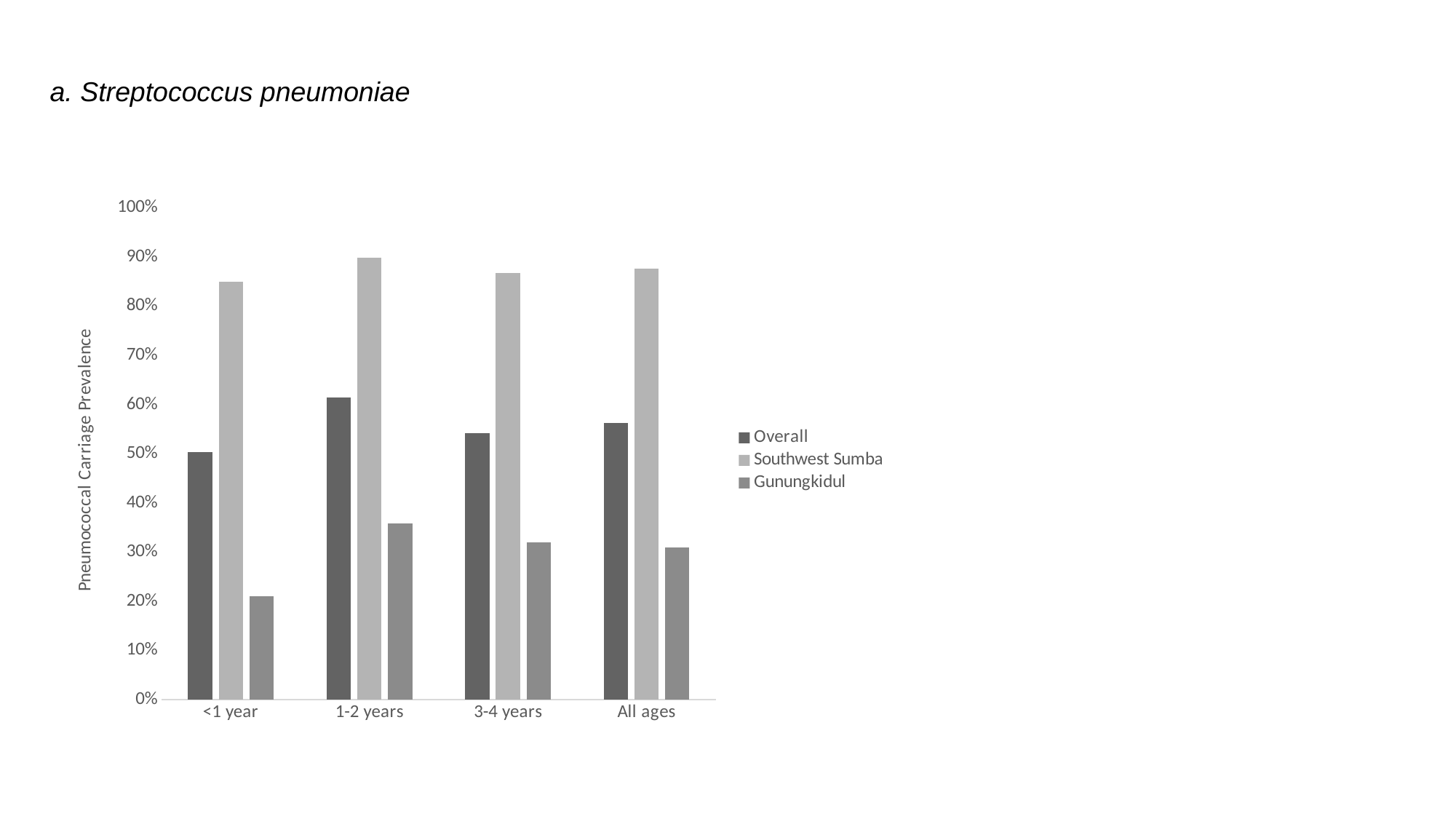

a. Streptococcus pneumoniae
### Chart
| Category | Overall | Southwest Sumba | Gunungkidul |
|---|---|---|---|
| <1 year | 0.503 | 0.85 | 0.21 |
| 1-2 years | 0.614 | 0.898 | 0.359 |
| 3-4 years | 0.542 | 0.867 | 0.32 |
| All ages | 0.563 | 0.876 | 0.309 |

## Slide 2
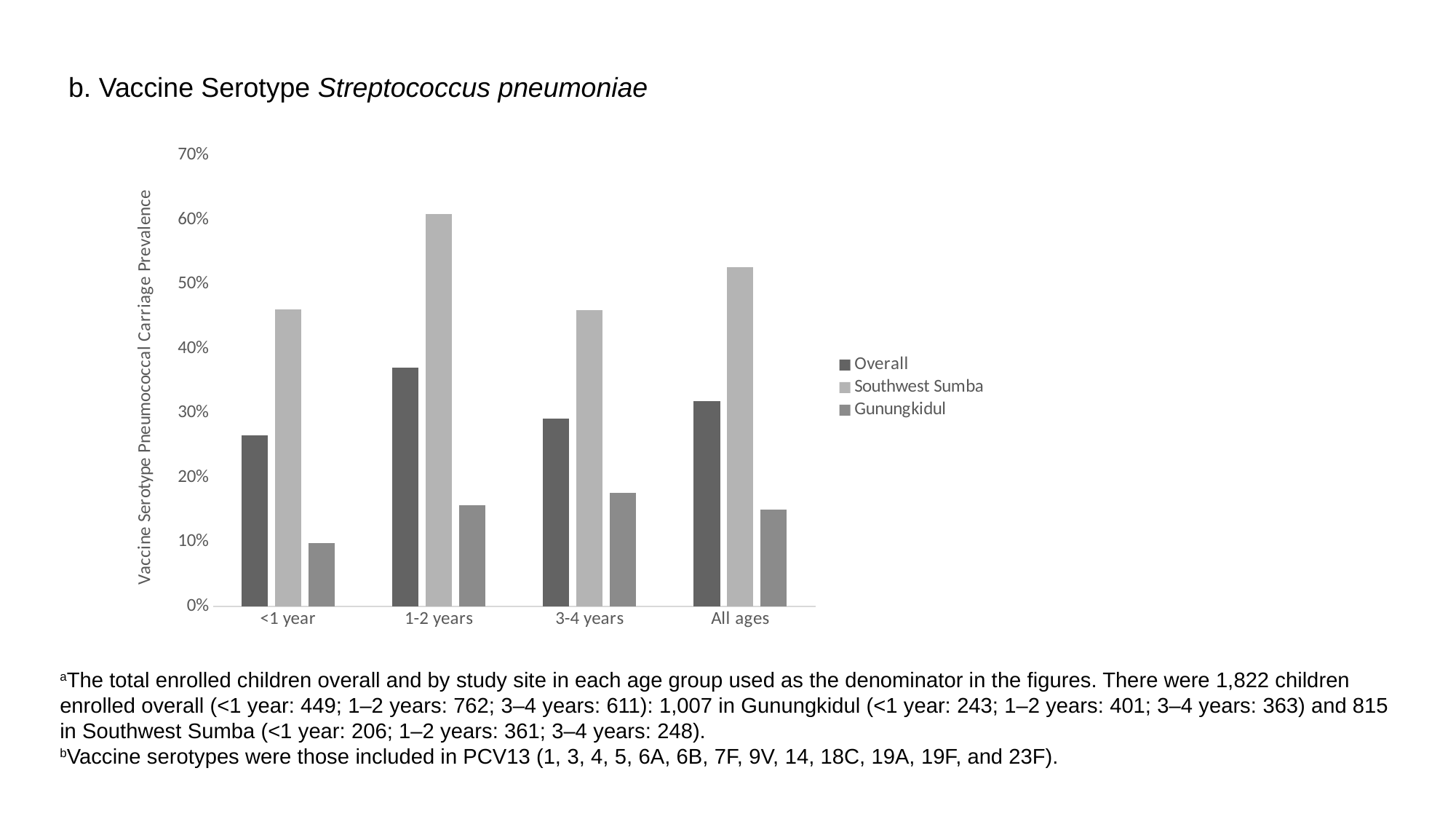

b. Vaccine Serotype Streptococcus pneumoniae
### Chart
| Category | Overall | Southwest Sumba | Gunungkidul |
|---|---|---|---|
| <1 year | 0.265 | 0.461 | 0.0987 |
| 1-2 years | 0.371 | 0.609 | 0.1571 |
| 3-4 years | 0.291 | 0.46 | 0.176 |
| All ages | 0.318 | 0.526 | 0.15 |aThe total enrolled children overall and by study site in each age group used as the denominator in the figures. There were 1,822 children enrolled overall (<1 year: 449; 1–2 years: 762; 3–4 years: 611): 1,007 in Gunungkidul (<1 year: 243; 1–2 years: 401; 3–4 years: 363) and 815 in Southwest Sumba (<1 year: 206; 1–2 years: 361; 3–4 years: 248).
bVaccine serotypes were those included in PCV13 (1, 3, 4, 5, 6A, 6B, 7F, 9V, 14, 18C, 19A, 19F, and 23F).
