# Supplementary material for: Nasopharyngeal carriage of Streptococcus pneumoniae among children <5 years of age in Indonesia prior to pneumococcal conjugate vaccine introduction
Source: PLoS One. 2024 Jan 11;19(1):e0297041. doi: 10.1371/journal.pone.0297041 (PMC10783721; doi:10.1371/journal.pone.0297041)
Supplement: S1 File — (PDF) [file pone.0297041.s004.pdf]

	Interpretive Categories and MIC Breakpoints (μg/mL)		
**Antibiotic Class**	**S**	**I**	**R**
**Cephalosporins**			
Cefepime			
Non-meningitis breakpoints	≤1	2	≥4
Meningitis breakpoints	≤0.5	1	≥2
Ceftriaxone			
Non-meningitis breakpoints	≤1	2	≥4
Meningitis breakpoints	≤0.5	1	≥2
**Penicillins**			
Penicillin			
Non-meningitis breakpoints	≤2	4	≥8
Meningitis breakpoints	≤0.06	-	≥0.12

MIC: minimum inhibitory concentration; S: susceptible; I: intermediate; R: resistant

[a]According to the 2022 Clinical and Laboratory Standards Institute guidelines [14].
