# Supplementary material for: Nasopharyngeal carriage of Streptococcus pneumoniae among children <5 years of age in Indonesia prior to pneumococcal conjugate vaccine introduction
Source: PLoS One. 2024 Jan 11;19(1):e0297041. doi: 10.1371/journal.pone.0297041 (PMC10783721; doi:10.1371/journal.pone.0297041)
Supplement: S2 File — (PDF) [file pone.0297041.s005.pdf]

Serotype	Gunungkidul				Southwest Sumba				Total			
	Susceptible[b]		NS to one or more antibiotic[c]		Susceptible[b]		NS to one or more antibiotic[c]		Susceptible[b]		NS to one or more antibiotic[c]	
	n/N	%	n/N	%	n/N	%	n/N	%	n/N	%	n/N	%
**1[a]**	-	-	-	-	0/1	0.0	1/1	100.0	0/1	0.0	1/1	100.0
**3[a]**	9/15	60.0	6/15	40.0	10/15	66.7	5/15	33.3	19/30	63.3	11/30	36.7
**4[a]**	0/1	0.0	1/1	100.0	0/3	0.0	3/3	100.0	0/4	0.0	4/4	100.0
**6A[a]**	1/2	50.0	1/2	50.0	7/35	20.0	28/35	80.0	8/37	21.6	29/37	78.4
**6B[a]**	13/53	24.5	40/53	75.5	12/138	8.7	126/138	91.3	25/191	13.1	166/191	86.9
**7F[a]**	0/1	0.0	1/1	100.0	0/3	0.0	3/3	100.0	0/4	0.0	4/4	100.0
**9V[a]**	-	-	-	-	2/2	100.0	0/2	0.0	2/2	100.0	0/2	0.0
**14[a]**	1/10	10.0	9/10	90.0	4/36	11.1	32/36	88.9	5/46	10.9	41/46	89.1
**18C[a]**	1/5	20.0	4/5	80.0	6/8	75.0	2/8	25.0	7/13	53.8	6/13	46.2
**19A[a]**	0/3	0.0	3/3	100.0	3/48	6.3	45/48	93.8	3/51	5.9	48/51	94.1
**19F[a]**	2/51	3.9	49/51	96.1	33/86	38.4	53/86	61.6	35/137	25.5	102/137	74.5
**23F[a]**	3/13	23.1	10/13	76.9	50/73	68.5	23/73	31.5	53/86	61.6	33/86	38.4
**2**	-	-	-	-	1/1	100.0	0/1	0.0	1/1	100.0	0/1	0.0
**6C**	31/36	86.1	5/36	13.9	11/19	57.9	8/19	42.1	42/55	76.4	13/55	23.6
**6D**	-	-	-	-	0/5	0.0	5/5	100.0	0/5	0.0	5/5	100.0
**7C**	2/3	66.7	1/3	33.3	4/8	50.0	4/8	50.0	6/11	54.5	5/11	45.5
**8**	1/1	100.0	0/1	0.0	0/3	0.0	3/3	100.0	1/4	25.0	3/4	75.0
**9A**	-	-	-	-	2/2	100.0	0/2	0.0	2/2	100.0	0/2	0.0
**9N**	-	-	-	-	1/1	100.0	0/1	0.0	1/1	100.0	0/1	0.0
**10A**	4/4	100.0	0/4	0.0	6/8	75.0	2/8	25.0	10/12	83.3	2/12	16.7
**10B**	1/2	50.0	1/2	50.0	1/1	100.0	0/1	0.0	2/3	66.7	1/3	33.3
**10F**	-	-	-	-	3/3	100.0	0/3	0.0	3/3	100.0	0/3	0.0

**11A**	0/4	0.0	4/4	100.0	4/40	10.0	36/40	90.0	4/44	9.1	40/44	90.9
**11D**	1/1	100.0	0/1	0.0	-	-	-	-	1/1	100.0	0/1	0.0
**12F**	-	-	-	-	0/1	0.0	1/1	100.0	0/1	0.0	1/1	100.0
**13**	4/6	66.7	2/6	33.3	8/21	38.1	13/21	61.9	12/27	44.4	15/27	55.6
**15A**	0/3	0.0	3/3	100.0	1/7	14.3	6/7	85.7	1/10	10.0	9/10	90.0
**15B**	3/9	33.3	6/9	66.7	12/18	66.7	6/18	33.3	15/27	55.6	12/27	44.4
**15C**	6/11	54.5	5/11	45.5	7/13	53.8	6/13	46.2	13/24	54.2	11/24	45.8
**16F**	7/9	77.8	2/9	22.2	5/11	45.5	6/11	54.5	12/20	60.0	8/20	40.0
**17F**	1/1	100.0	0/1	0.0	3/3	100.0	0/3	0.0	4/4	100.0	0/4	0.0
**18A**	-	-	-	-	2/2	100.0	0/2	0.0	2/2	100.0	0/2	0.0
**18B**	-	-	-	-	1/2	50.0	1/2	50.0	1/2	50.0	1/2	50.0
**18F**	-	-	-	-	0/1	0.0	1/1	100.0	0/1	0.0	1/1	100.0
**19B**	-	-	-	-	1/3	33.3	2/3	66.7	1/3	33.3	2/3	66.7
**20**	-	-	-	-	4/5	80.0	1/5	20.0	4/5	80.0	1/5	20.0
**21**	-	-	-	-	5/6	83.3	1/6	16.7	5/6	83.3	1/6	16.7
**22F**	1/2	50.0	1/2	50.0	4/4	100.0	0/4	0.0	5/6	83.3	1/6	16.7
**23A**	2/2	100.0	0/2	0.0	4/7	57.1	3/7	42.9	6/9	66.7	3/9	33.3
**23B**	0/1	0.0	1/1	100.0	1/2	50.0	1/2	50.0	1/3	33.3	2/3	66.7
**25A**	1/1	100.0	0/1	0.0	-	-	-	-	1/1	100.0	0/1	0.0
**27**	-	-	-	-	1/1	100.0	0/1	0.0	1/1	100.0	0/1	0.0
**28F**	-	-	-	-	1/1	100.0	0/1	0.0	1/1	100.0	0/1	0.0
**31**	-	-	-	-	0/1	0.0	1/1	100.0	0/1	0.0	1/1	100.0
**34**	14/22	63.6	8/22	36.4	8/12	66.7	4/12	33.3	22/34	64.7	12/34	35.3
**33B**	-	-	-	-	2/3	66.7	1/3	33.3	2/3	66.7	1/3	33.3
**35A**	2/6	33.3	4/6	66.7	3/3	100.0	0/3	0.0	5/9	55.6	4/9	44.4
**35B**	1/4	25.0	3/4	75.0	4/13	30.8	9/13	69.2	5/17	29.4	12/17	70.6
**35C**	1/2	50.0	1/2	50.0	-	-	-	-	1/2	50.0	1/2	50.0
**35F**	0/2	0.0	2/2	100.0	1/2	50.0	1/2	50.0	1/4	25.0	3/4	75.0

**36**	1/1	100.0	0/1	0.0	-	-	-	-	1/1	100.0	0/1	0.0
**37**	0/1	0.0	1/1	100.0	-	-	-	-	0/1	0.0	1/1	100.0
**38**	0/1	0.0	1/1	100.0	-	-	-	-	0/1	0.0	1/1	100.0
**39**	-	-	-	-	0/4	0.0	4/4	100.0	0/4	0.0	4/4	100.0
**42**	-	-	-	-	4/4	100.0	0/4	0.0	4/4	100.0	0/4	0.0
**Non-typeable**	23/34	67.6	11/34	32.4	47/95	49.5	48/95	50.5	70/129	54.3	59/129	45.7

NS: non-susceptibility

[a]PCV13-type (1, 3, 4, 5, 6A, 6B, 7F, 9V, 14, 18C, 19A, 19F, and 23F)

[b]Susceptible to all antibiotic classes tested.

[c]Isolates with intermediate or resistant to ≥1 antibiotic were classified as NS.
