# Supplementary material for: Nasopharyngeal carriage of Streptococcus pneumoniae among children <5 years of age in Indonesia prior to pneumococcal conjugate vaccine introduction
Source: PLoS One. 2024 Jan 11;19(1):e0297041. doi: 10.1371/journal.pone.0297041 (PMC10783721; doi:10.1371/journal.pone.0297041)
Supplement: S3 File — (PDF) [file pone.0297041.s006.pdf]

Serotype	Gungunkidul		Southwest Sumba		Total	
	MDNS[b]		MDNS[b]		MDNS[b]	
	n/N	%	n/N	%	n/N	%
**1[a]**	-	-	1/1	100.0	1/1	100.0
**3[a]**	0/15	0.0	0/15	0.0	0/30	0.0
**4[a]**	0/1	0.0	0/3	0.0	0/4	0.0
**6A[a]**	0/2	0.0	2/35	5.7	2/37	5.4
**6B[a]**	9/53	17.0	13/138	9.4	22/191	11.5
**7F[a]**	0/1	0.0	0/3	0.0	0/4	0.0
**9V[a]**	-	-	0/2	0.0	0/2	0.0
**14[a]**	1/10	10.0	2/36	5.6	3/46	6.5
**18C[a]**	0/5	0.0	0/8	0.0	0/13	0.0
**19A[a]**	1/3	33.3	12/48	25.0	13/51	25.5
**19F[a]**	41/51	80.4	29/86	33.7	70/137	51.1
**23F[a]**	4/13	30.8	5/73	6.8	9/86	10.5
**6D**	-	-	1/5	20.0	1/5	20.0
**11A**	0/4	0.0	2/40	5.0	2/44	4.5
**13**	0/6	0.0	9/21	42.9	9/27	33.3
**15C**	0/11	0.0	1/13	7.7	1/24	4.2
**16F**	0/9	0.0	1/11	9.1	1/20	5.0
**22F**	1/2	50.0	0/4	0.0	1/6	16.7
**34**	1/22	4.5	0/12	0.0	1/34	2.9
**35A**	2/6	33.3	0/3	0.0	2/9	22.2
**Non-typeable**	3/34	8.8	6/95	6.3	9/129	7.0

MDNS: multi-drug non-susceptiblity

[a]PCV13-type (1, 3, 4, 5, 6A, 6B, 7F, 9V, 14, 18C, 19A, 19F, and 23F)
[b]MDNS was defined as non-susceptiblity to ≥3 classes of antibiotics.
